# Human cytomegalovirus induces apoptosis in neural stem/progenitor cells derived from induced pluripotent stem cells by generating mitochondrial dysfunction and endoplasmic reticulum stress

**DOI:** 10.1186/2042-4280-4-2

**Published:** 2013-10-21

**Authors:** Hiroyuki Nakamura, Huanan Liao, Kahori Minami, Masashi Toyoda, Hidenori Akutsu, Yoshitaka Miyagawa, Hajime Okita, Nobutaka Kiyokawa, Akihiro Umezawa, Ken-Ichi Imadome, Naoki Inoue, Shigeyoshi Fujiwara

**Affiliations:** 1 Department of Infectious Diseases, National Research Institute for Child Health and Development, 2-10-1 Okura, Setagaya-ku, Tokyo 157-8535, Japan; 2 Department of Reproductive Biology, Center for Regenerative Medicine, National Research Institute for Child Health and Development, 2-10-1 Okura, Setagaya-ku, Tokyo 157-8535, Japan; 3 Department of Pediatric Hematology and Oncology Research, National Research Institute for Child Health and Development, 2-10-1 Okura, Setagaya-ku, Tokyo 157-8535, Japan; 4 Department of Virology I, National Institute of Infectious Diseases, 1-23-1 Toyama, Shinjuku-ku, Tokyo 162-8640, Japan

**Keywords:** Human cytomegalovirus, iPS cells, Neural stem/progenitor cells, Apoptosis, ER stress

## Abstract

**Background:**

Congenital human cytomegalovirus (HCMV) infection, a leading cause of birth defects, is most often manifested as neurological disorders. The pathogenesis of HCMV-induced neurological disorders is, however, largely unresolved, primarily because of limited availability of model systems to analyze the effects of HCMV infection on neural cells.

**Methods:**

An induced pluripotent stem cell (iPSC) line was established from the human fibroblast line MRC5 by introducing the Yamanaka’s four factors and then induced to differentiate into neural stem/progenitor cells (NSPCs) by dual inhibition of the SMAD signaling pathway using Noggin and SB-431542.

**Results:**

iPSC-derived NSPCs (NSPC/iPSCs) were susceptible to HCMV infection and allowed the expression of both early and late viral gene products. HCMV-infected NSPC/iPSCs underwent apoptosis with the activation of caspase-3 and −9 as well as positive staining by the terminal deoxynucleotidyl transferase-mediated dUTP nick-end labeling (TUNEL). Cytochrome c release from mitochondria to cytosol was observed in these cells, indicating the involvement of mitochondrial dysfunction in their apoptosis. In addition, phosphorylation of proteins involved in the unfolded protein response (UPR), such as PKR-like eukaryotic initiation factor 2a kinase (PERK), c-Jun NH2-terminal kinase (JNK), inositol-requiring enzyme 1 (IRE1), and the alpha subunit of eukaryotic initiation factor 2 (eIF2α) was observed in HCMV-infected NSPC/iPSCs. These results, coupled with the finding of increased expression of mRNA encoding the C/EBP-homologous protein (CHOP) and the detection of a spliced form of X-box binding protein 1 (XBP1) mRNA, suggest that endoplasmic reticulum (ER) stress is also involved in HCMV-induced apoptosis of these cells.

**Conclusions:**

iPSC-derived NSPCs are thought to be a useful model to study HCMV neuropathogenesis and to analyze the mechanisms of HCMV-induced apoptosis in neural cells.

## Background

Congenital cytomegalovirus (CMV) infection is a major cause of birth defects resulting mainly from primary CMV infection during pregnancy. At birth, approximately 5 to 10% of congenitally infected newborns are estimated to be symptomatic exhibiting multi-organ disorders including neurological defects such as mental retardation, sensorineural hearing loss, and microencephaly [1,2]. A latest study showed that if laboratory findings including those from magnetic resonance imaging (MRI) images of the brain are considered, up to 30% of congenitally infected newborns exhibit some abnormal signs [3]. Sixty to 90% of congenitally infected children who are symptomatic at birth, and 10 to 15% of those who are asymptomatic at birth develop one or more long-term sequelae. Although CMV infects a wide variety of cell types, infection of the nervous system gives most serious and long-lasting damages to the host.

As a part of understanding the HCMV neuropathogenesis, it is important to scrutinize the cellular response to CMV infection in neural cells. Some human neural cell lines can be infected with HCMV with different permissiveness to HCMV gene expression and replication [4-7]. A recent study has shown that neural progenitor cells isolated from developing human brain tissues are susceptible to CMV infection and undergo apoptosis following infection [8,9]. However, the amount of neural cells obtainable from human brain tissues is limited.

Pluripotent stem cells, including embryonic stem cells (ESCs) and induced pluripotent stem cells (iPSCs), are characterized by the ability to differentiate into tissues derived from any of the three embryonic germ layers. Recent advances in the method to induce efficient differentiation of either ESCs or iPSCs into specific cell lineages offer an opportunity to establish model systems for viral infections of various cell types, including neural cells. Furthermore, differentiated cells derived from pluripotent stem cells are obtainable in potentially unlimited amounts. Previous works revealed that while mouse ESCs are not susceptible to murine CMV (MCMV), NSPCs that are differentiated from them are susceptible and their proliferation and differentiation are suppressed by MCMV [10-13]. Experiments with human ESCs are, however, complicated with ethical problems.

In this study, to analyze the pathological effects of HCMV on neural cells, we prepared NSPCs from human iPSCs and examined whether NSPCs are susceptible to HCMV infection. The results indicated that NSPCs are susceptible to HCMV infection and undergo apoptosis caused by mitochondrial dysfunction and endoplasmic reticulum (ER) stress.

## Methods

### Cells and viruses

The human fetal lung fibroblast MRC5 was grown in Dulbecco’s modified Eagle’s medium (DMEM) supplemented with 10% fetal bovine serum (FBS; Invitrogen, Carlsbad, CA). The human foreskin fibroblast cell line hTERT-BJ1 immortalized with the human telomerase reverse transcriptase (Clontech, Palo Alto, CA) was grown in a medium consisting of 4 parts of DMEM and 1 part of medium 199 (Sigma) supplemented with 10% FBS, 1 mM sodium pyruvate (Sigma), and 2 mM glutamine (Invitrogen). HCMV laboratory strain Towne (ATCC VR-977) was propagated in hTERT-BJ1 cells. The human iPSC line MRC-iPS-25 that was established from MRC5 by retroviral vector-mediated transduction of the c-Myc, Oct-4, Klf4, and Sox2 genes [14,15] were cultured on mitomycin C-treated mouse embryonic fibroblasts (MEFs) in an iPSC medium consisting of Knockout DMEM/F12 (Invitrogen) supplemented with non-essential amino acids (0.1 mM, Invitrogen), glutamax I (1 mM, Invitrogen), 20% Knockout Serum Replacement (Invitrogen), β-mercaptoethanol (55 μM, Invitrogen) and basic fibroblast growth factor (10 ng/mL; Peprotech, Rocky Hill, NJ).

### Induced differentiation on iPSCs into neural stem cells

MRC-iPSC-25 cells cultured under feeder-free conditions were induced to differentiate into neural stem/progenitor cells (NSPCs) by the method of dual inhibition of the SMAD signaling pathway described previously [16]. In brief, feeder-free iPSCs were treated with the mTeSR1 medium (StemCell Technologies, Vancouver, BC, Canada) containing Y27632 (Wako Pure Chemicals, Osaka, Japan) and maintained with a daily medium change for 4 days. Then the medium was replaced with iPSC medium supplemented with SB431542 (10 nM, Wako Pure Chemicals) and Noggin (500 ng/ml, Wako Pure Chemicals). This date was designated day 0. On day 2, culture medium was replaced with a medium consisting of 3 parts of iPSC medium and 1 part of N2 medium (Knockout DMEM/F12 containing 1× N2 supplement) supplemented with SB431542 (10 nM) and Noggin (500 ng/ml). On day 4, culture medium was replaced with a medium consisting of 1 part of iPSC medium and 1 part of N2 medium supplemented with SB431542 (10 nM) and Noggin (500 ng/ml). On day 6, cells were expanded in StemPro NSC SFM (Invitrogen). MRC-iPSC-25 cells cultured under feeder-free conditions and NSPC/iPSCs were infected with the Towne strain HCMV at a multiplicity of infection (MOI) of 1 plaque forming unit (PFU) per cell. To detect infectious virions produced from HCMV-infected NSPC/iPSCs, supernatant was collected and replaced with fresh medium every two days after infection. hTERT-BJ1 cells were inoculated with the supernatant and examined by IFA for expression of IE1/IE2.

### Antibodies

Antibodies used were as follows: rabbit anti-Sox2, rabbit anti-Nanog, rabbit anti-Oct-4, rabbit anti-cleaved caspase-3, rabbit anti-cleaved caspase-9, rabbit anti-phospho-eIF2α (Ser51), rabbit anti-phospho-PERK (Thr980), and rabbit anti-phospho-SAPK/JNK (Thr183/Tyr185)(Cell Signaling Technology, Beverly, MA); mouse anti-CMV IE1/IE2, rabbit anti-Musashi-1, and rabbit anti-cytochrome c (Millipore, Temecula, CA); rabbit anti-Nestin and mouse anti-α-tubulin (Sigma-Aldrich, St. Louis, MO); rabbit anti-Pax6 (Covance, Princeton, NJ), mouse anti-CMV gB (Abcam, Cambridge, MA); mouse anti-pp65 (Virusys Corporation, Sykesville, MD); rabbit anti-phosphorylated IRE1α (Pierce/Thermo Scientific, Rockford, IL); Alexa Fluor 488-conjugated goat anti-mouse IgG and Alexa Fluor 594-conjugated goat anti-rabbit IgG (Molecular Probes, Eugene, OR); horseradish peroxidase-conjugated donkey anti-rabbit IgG and horseradish peroxidase-conjugated sheep anti-mouse IgG (GE Healthcare, UK).

### Immunofluorescence microscopy and immunoblot analysis

Cells were fixed with 4% parafolmaldehyde in PBS (Wako chemicals) at room temperature (RT) for 15 min. After fixation, cells were treated with 1.0% Triton X-100 in PBS for 15 min at RT and blocked with 10% goat serum in PBS for 30 min. Cells were incubated with the primary antibody at 4°C overnight, followed by washing in PBS and incubation with the corresponding secondary antibody at 37°C for 1 h. Nuclei were stained with DAPI. For the assessment of cell death, terminal deoxynucleotidyl transferase (TdT)-mediated dUTP nick-end labeling (TUNEL) assay was performed according to the manufacturer’s instructions (Roche). Immunoblot analyses were performed as described previously [17].

### Reverse transcriptase (RT)-PCR and real-time quantitative RT-PCR

Total RNA was isolated from mock- or HCMV-infected cells using TRIzol reagent (Invitrogen). Reverse transcription was performed on each RNA sample (5 μg) using SuperScript III First-Strand Synthesis System for RT-PCR (Invitrogen). Primer sequences are shown in Table 1. RT-PCR products were resolved by electrophoresis on 2% agarose gel and then visualized by ultraviolet illumination after ethidium bromide staining. Real-time quantitative RT-PCR was performed using TaqMan^TM^ Universal Master Mix II with UNG (Applied Biosystems) on a StepOne Plus PCR System (Applied Biosystems). Amplifications were achieved in a final volume of 25 μl containing TaqMan probes labeled with FAM on the 5’-end and MGB on the 3’-end. The primers and probes for *UL136* were: forward primer, 5’-GGCCGTTGAACGGAGCTAT-3’ and reverse primer, 5’-CCATTTCCACCGTGTCGAA-3’, and TaqMan probe, 5’-FAM-TACTACGGCAGCGGCT-MGB-3’. The forward and reverse primers and reporter probes for HCMV *IE1*, *UL89*, and Human *G6PD* were described previously [18].

**Table 1:** List of primer sequences for RT-PCR

**Gene**	**Forward primer**	**Reverse primer**
IE1*	ATGGAGTCCTCTGCCAAGAG	ATTCTATGCCGCACCATGTCC
IE2*	ATGGAGTCCTCTGCCAAGAG	CTGAGACTTGTTCCTCAGGTCCTG
vIL-10*	ATGCTGTCGGTGATGGTCTCTTCC	CTTTCTCGAGTGCAGATACTCTTCG
UL36*	GACCTACGGGACACGCTGATG	TGTGGAAGTGGTCGCAGTGAC
UL38	GACTACGACCACGCATAGCA	GGGAACAGAGCGTTCCAATA
pp65	CGCAACCTGGTGCCCATGG	CGTTTGGGTTGCGCAGCGGG
Nanog*	GCTTGCCTTGCTTTGAAGCA	TTCTTGACCGGGACCTTGTC
Oct-4	GAGCAAAACCCGGAGGAGT	TTCTCTTTCGGGCCTGCAC
Sox1	GCGGAAAGCGTTTTCTTTG	TAATCTGACTTCTCCTCCC
Sox2	ATGCACCGCTACGACGTGA	CTTTTGCACCCCTCCCATTT
Pax6*	AACAGACACAGCCCTCACAAACA	CGGGAACTTGAACTGGAACTGAC
Nestin*	CAGCGTTGGAACAGAGGTTGG	TGGCACAGGTGTCTCAAGGGTAG
MAP2*	CCACCTGAGATTAAGGATCA	GGCTTACTTTGCTTCTCTGA
GFAP*	GTACCAGGACCTGCTCAAT	CAACTATCCTGCTTCTGCTC
OSP*	ACTGCTGCTGACTGTTCTTC	GTAGAAACGGTTTTCACCAA
XBP1*	CCTTGTAGTTGAGAACCAGG	GGGGCTTGGTATATATGTGG
CHOP*	TGGAAGCCTGGTATGAGGAC	TCACCATTCGGTCAATCAGA
β-actin*	ACCATGGATGATGATATCGC	TCATTGTAGAAGGTGTGGTG
GAPDH*	CCACCCATGGCAAATTCCATGGCA	TCTAGACGGCAGGTCAGGTCCACC

Asterisks (*) indicate that amplified fragments contain splicing junctions. Amplified fragments for UL38, pp65, Oct-4, Sox1, and Sox2 did not contain splicing junctions, and therefore control experiments without reverse transcriptase confirmed the RNA origin of the PCR products.

## Results

### Preparation of human iPSC-derived neural stem/progenitor cells

**Figure 1:**
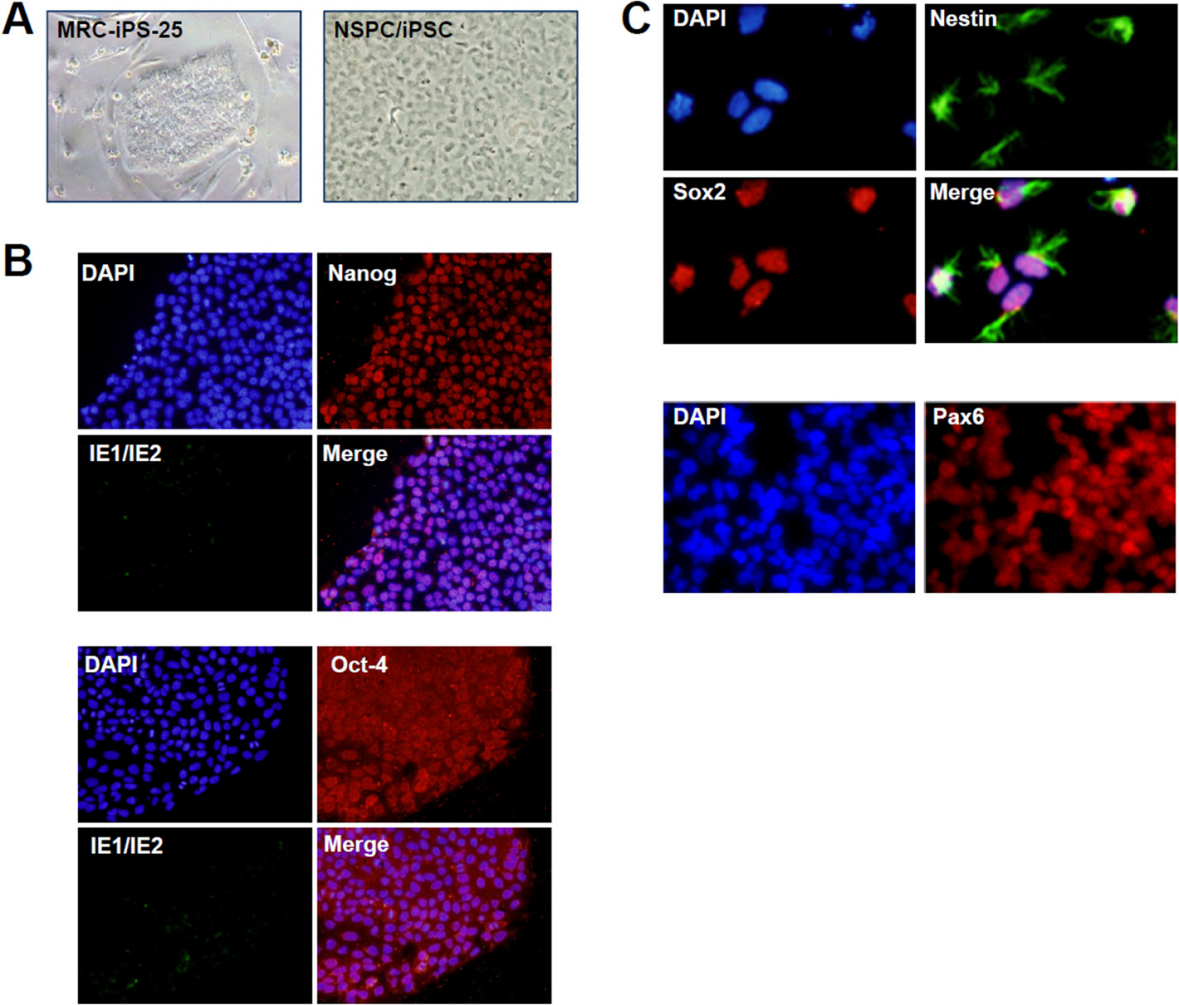
**Differentiation of MRC-iPS-25 cells to neural stem/progenitor cells. (A)** Phase-contrast images of MRC-iPS-25 cells cultured on a feeder layer of MEFs (left) and NSPC/iPSCs (right). **(B)** Immunofluorescence analysis of Towne-infected MRC-iPS-25 cells cultured under feeder-free conditions at 2 dpi stained with primary antibodies for pluripotent markers (Nanog or Oct-4) and HCMV IE1/IE2 proteins. Antigen proteins were detected with Alexa Fluor 488-conjugated goat anti-mouse IgG or Alexa Fluor 594-conjugated goat anti-rabbit IgG antibody. Nuclei were stained with DAPI. **(C)** Immunofluorescence analysis of NSPC markers Nestin, Sox2, and Pax6 in NSPC/iPSCs. NSPC/iPSCs were fixed and reacted with anti-Nestin (green), anti-Sox2 (red), and anti-Pax6 (red) antibodies, followed by detection with secondary antibodies. Immunofluorescence signals were obtained using a fluorescence microscope IX71. Representative results from three independent experiments are shown.

Figure 1A demonstrates that MRC-iPS-25 cells have a typical iPSC colony morphology. The expression of pluripotency markers of iPSCs such as Nanog and Oct-4 in MRC-iPS-25 cells was confirmed by indirect immunofluorescence assay (IFA) (Figure 1B). The HCMV-encoded proteins IE1/IE2 were not detected in MRC-iPS-25 cells following inoculation with the virus, indicating that MRC-iPS-25 cells are either not susceptible to HCMV infection or do not support expression of the IE genes (Figure 1B).

NSPC/iPSCs prepared by induced differentiation of MRC-iPS-25 cells proliferated in a monolayer and displayed a rounded, immature neural morphology (Figure 1A). IFA (Figure 1C) showed that NSPC/iPSCs expressed the NSC markers Nestin, Sox2, and Pax6, indicating that NSPC/iPSCs have the authentic NSPC phenotype.

### In vitro HCMV infection of iPSC-derived NSPCs

**Figure 2:**
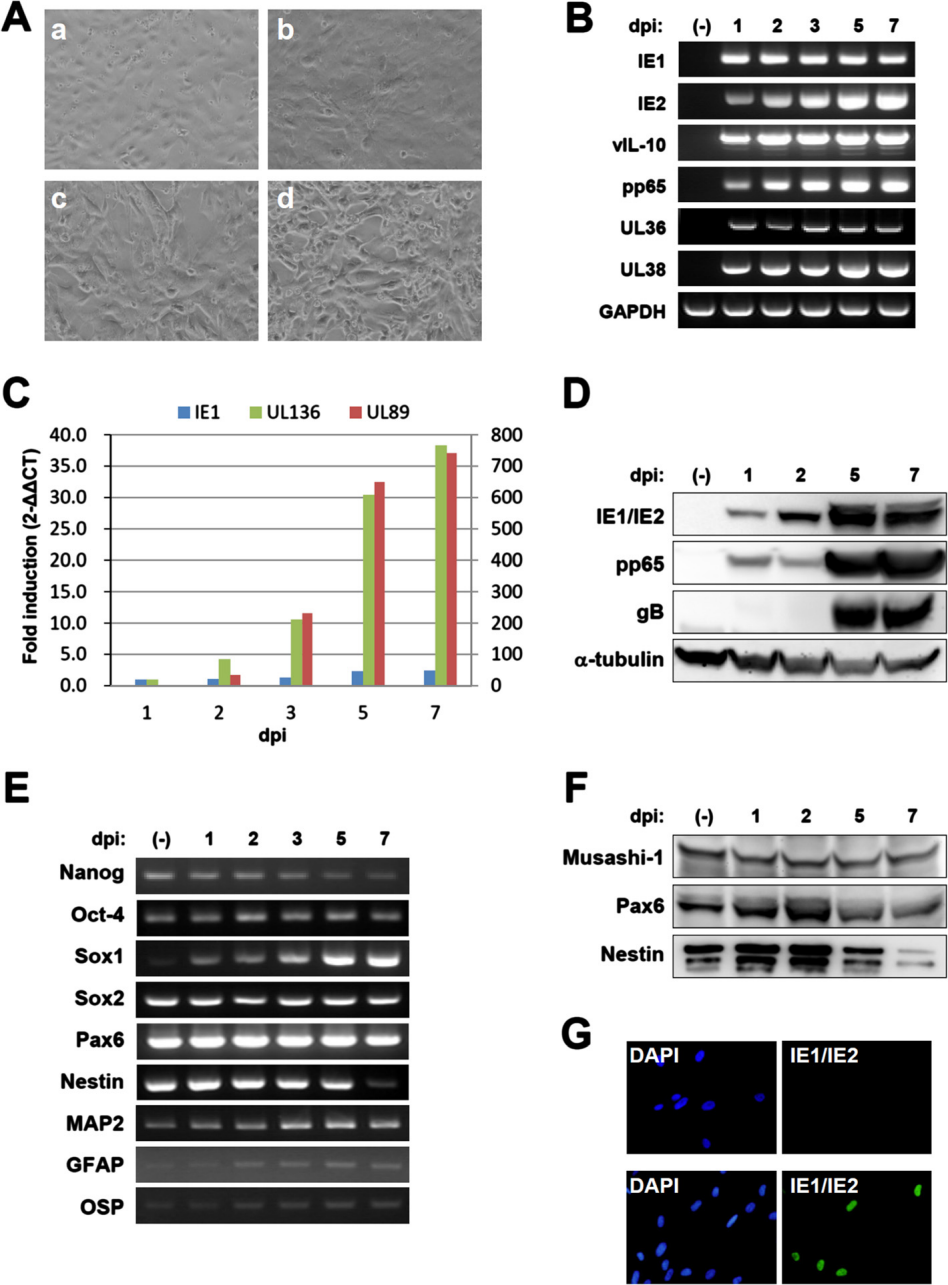
**Analysis on the expression of viral and cellular gene products in NSPC/iPSCs. (A)** Morphological changes of Towne-infected NSPC/iPSCs were observed under the inverted microscope before infection **(a)**, 2 dpi **(b)**, 5 dpi **(c)**, and 7 dpi **(d)**. **(B)** RT-PCR analysis of HCMV-encoding gene expression. Total RNAs isolated from NSPC/iPSCs harvested before (−) HCMV infection or at 1, 2, 3, 5, and 7 dpi with HCMV Towne strain were subjected to RT-PCR assays. GAPDH gene expression was assayed for the control. **(C)** The kinetics of mRNA expression for IE1, UL89, and UL136 in Towne-infected NSPC/iPSCs was examined by real-time quantitative RT-PCR assay. The mRNA expression was normalized to that of G6PDH gene. Real-time PCR data was analyzed by the 2-ΔΔCT method. The fold induction was calculated as the ratio of mRNA levels detected at each time point to that detected at 1 dpi. The y-axis represents fold induction of IE1 and UL136 mRNA (left y-axis) and UL89 mRNA (right y-axis). **(D)** Immunoblot analysis of HCMV protein expression in HCMV-infected NSPC/iPSCs. Whole-cell lysates of NSPC/iPSCs harvested before (−) HCMV infection or at 1, 2, 5, and 7 dpi with HCMV Towne strain were separated by SDS-PAGE and analyzed by immunoblotting with antibodies against IE1/IE2, pp65, gB, and α-tubulin. **(E)** RT-PCR analysis of pluripotency and neural differentiation marker gene expression in HCMV-infected NSPC/iPSCs. **(F)** Immunoblot analysis of neural differentiation marker protein expression in HCMV-infected NSPC/iPSCs. Whole-cell lysates of NSPC/iPSCs were analyzed by immunoblotting with antibodies against Musashi-1, Pax6, and Nestin. **(G)** hTERT-BJ1 cells inoculated with culture supernatant collected from mock-infected NSPC/iPSCs (upper panel) or Towne HCMV-infected NSPC/iPSCs (lower panel) at 8 dpi were subjected to immunofluorescence test with anti-IE1/IE2 antibody (green). Nuclei were stained with DAPI. Representative results from two independent experiments are shown.

To examine the susceptibility of NSPC/iPSCs to HCMV infection, these cells were inoculated in vitro with the HCMV Towne strain at an MOI of 1 PFU per cell (Figure 2A). On the second day post-infection (dpi), NSPC/iPSCs started to show morphological changes including increased cell volume and cell fusion, and the number of cells with these changes increased until 7 dpi (Figure 2A). To examine whether NSPC/iPSCs were capable of supporting HCMV gene expression, total RNA extracted from the infected NSPC/iPSCs was analyzed by RT-PCR. As shown in Figure 2B, mRNAs encoding IE1, IE2, vIL-10, and pp65 as well as those encoding HCMV anti-apoptotic proteins, such as UL36 and UL38, were detected. The kinetics of HCMV gene expression was analyzed by quantitative real-time RT-PCR (Figure 2C). IE1 mRNA was detected first on 1 dpi and increased steadily until 5 dpi. mRNAs for UL89 and UL136 were detected somewhat later and increased gradually until 7 dpi. The results showed the NSPC/iPSCs are susceptible to HCMV infection and allow the expression of several viral genes of both early and late functions.

Expression of HCMV genes in NSPC/iPSCs was evaluated at the protein level by immunoblot analysis on day 1, 2, 5, and 7 following HCMV infection. As shown in Figure 2D, the immediate-early protein IE1 was first detected at 1 dpi and its level increased until 5 dpi. Another immediate-early protein IE2 was detected a little later, becoming visible at 5 dpi. The expression of the HCMV lower matrix protein pp65 (ppUL83), already visible at 1 dpi, was markedly elevated at 5 and 7 dpi. The HCMV envelope glycoprotein B (gB; UL55) was detected at 5 to 7 dpi. Thus the expression of HCMV proteins of both immediate-early and late functions was demonstrated in NSPC/iPSCs.

We next examined the expression of cellular mRNAs encoding the pluripotency and neural differentiation markers (Figure 2E). Expression of the iPSC markers Nanog and Oct-4 remained at low levels following infection with HCMV, although that of Nanog tapered. While expression of the NSPC markers Sox2 and Pax6 were kept at high levels following HCMV infection, that of another NSPC marker Nestin was markedly suppressed at 7 dpi. In addition, expression of the neuronal marker microtubule-associated protein 2 (MAP2), the astrocyte marker glial fibrillary acidic protein (GFAP), and the oligodendrocyte marker oligodendrocyte-specific protein (OSP) was detected at low levels. Interestingly, Sox1, a marker specific to the neuroectodermal lineages [19], was markedly upregulated following infection with HCMV. Expression of the NSPC markers was evaluated also at the protein level by immunoblot analysis on 1, 2, 5, and 7 dpi (Figure 2F). In accordance with the results with RT-PCR, expression of Pax6 and Nestin was confirmed, and that of Nestin was found markedly decreased 7 dpi. Another NSPC marker Musashi-1 was also detected. To examine whether HCMV-infected NSPC/iPSCs produce infectious virions, culture supernatants collected from Towne HCMV-infected NSPC/iPSCs were inoculated to hTERT-BJ1 cells. Inoculated cells expressed IE1/IE2 indicating that infectious virions were produced from HCMV-infected NSPC/iPSCs (Figure 2G). The supernatant contained 30 PFU/mL of HCMV at 4, 6, 8 dpi, while no plaque forming virus was detected at 2 dpi.

### HCMV infection induces apoptosis in iPSC-derived NSPCs

**Figure 3:**
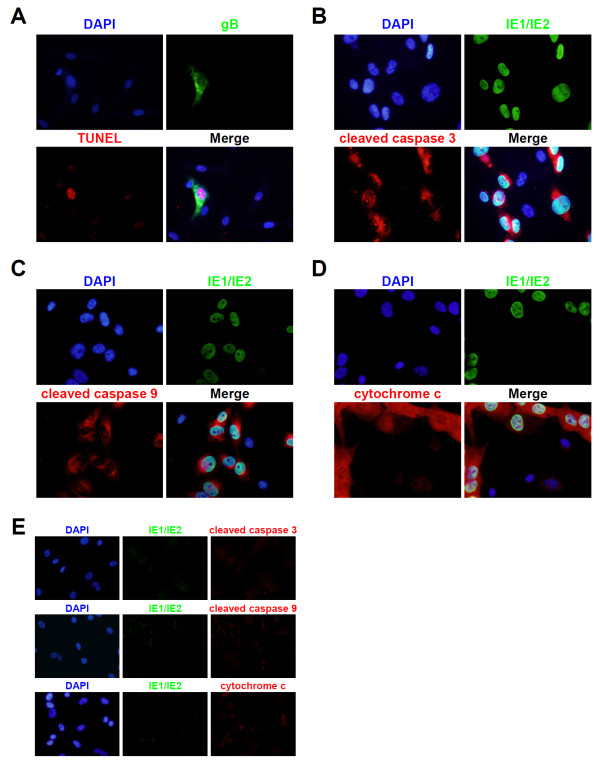
**HCMV-induced apoptosis of NSPC/iPSCs. (A)** Towne-infected NSPC/iPSCs at 6 dpi were subjected to TUNEL assay followed by immunofluorescence test with an anti-gB antibody. TUNEL-positive nuclei were stained in red. The anti-gB antibody was detected with Alexa Fluor 488-conjugated goat anti-mouse IgG antibody (green). Nuclei were stained with DAPI. **(B-D)** Towne-infected NSPC/iPSCs at 3 dpi were subjected to immunofluorescence test with anti-IE1/IE2 antibody in combination with anti-cleaved caspase 3 **(B)**, anti-cleaved caspase 9 **(C)**, or anti-cytochrome c **(D)** antibody. Alexa Fluor 488-conjugated goat anti-mouse IgG (green) or Alexa Fluor 594-conjugated goat anti-rabbit IgG antibody (red) was used as a secondary antibody. Nuclei were stained with DAPI. **(E)** Mock-infected NSPC/iPSCs were subjected to immunofluorescence test with anti-IE1/IE2 antibody in combination with anti-cleaved caspase 3 (upper panel), anti-cleaved caspase 9 (middle panel), or anti-cytochrome c (lower panel) antibody. Nuclei were stained with DAPI. Representative results from two independent experiments are shown.

To examine whether HCMV infection in NSPC/iPSCs induces apoptotic responses, we performed the TUNEL assay combined with IFA using an antibody specific to HCMV gB. As shown in Figure 3A, NSPC/iPSCs expressing gB was positive for TUNEL staining and those without gB expression was consistently negative. We also performed IFA to analyze the activation status of caspases using antibodies specific to the activated forms of caspase-3 and caspase-9. The results show that the activated forms of caspase-3 and caspase-9 were specifically detected in more than 80% of HCMV-infected NSPC/iPSCs expressing IE1/IE2 proteins (Figure 3B and 3C), but not in mock-infected NSPC/iPSCs (Figure 3E). To see whether mitochondrial dysfunction is involved in the activation of caspase 9, intracellular distribution of cytochrome c was analyzed in HCMV-infected cells by IFA. As shown in Figure 3D and 3E, strong signals of cytochrome c were detected in the cytosol of cells expressing IE1/IE2 proteins, while only faint signals of cytochrome c were detected in cells not expressing IE1/IE2 proteins or in mock-infected cells. These results indicate that HCMV infection of NSPC/iPSCs activated apoptotic responses involving release of mitochondrial cytochrome c and serial activation of caspases.

### Unfolded protein response in HCMV-infected NSPC/iPSCs

**Figure 4:**
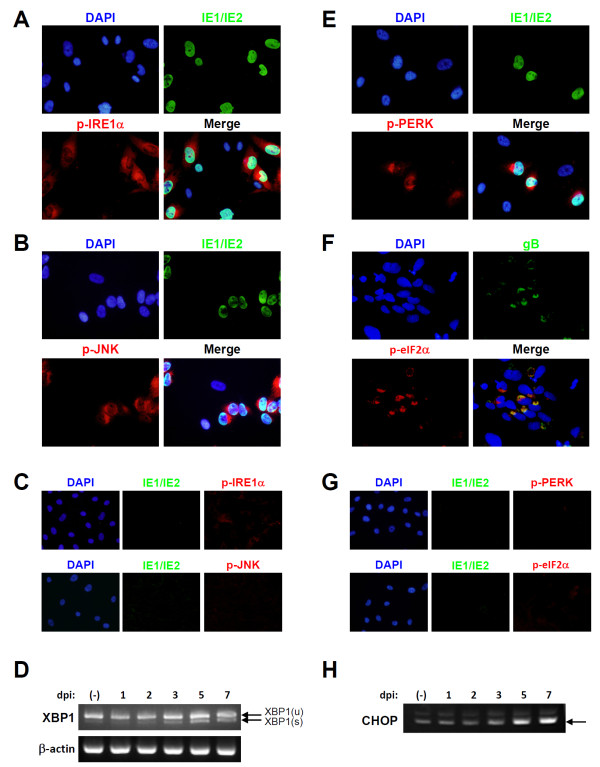
**HCMV-induced UPR in NSPC/iPSCs. (A and B)** Towne-infected NSPC/iPSCs at 3 dpi were subjected to immunofluorescence test with anti-IE1/IE2 antibody in combination with anti-phosphorylated IRE1α **(A)** or anti-phosphorylated JNK **(B)** antibody. Nuclei were stained with DAPI. **(C)** Mock-infected NSPC/iPSCs were subjected to immunofluorescence test with anti-IE1/IE2 antibody in combination with anti-phosphorylated IRE1α (upper panel) or anti-phosphorylated JNK (lower panel) antibody. Nuclei were stained with DAPI. **(D)** Detection of XBP1 (u, unspliced) and XBP1 (s, spliced) mRNAs in HCMV-infected NSPC/iPSCs. Total RNAs isolated from NSPC/iPSCs harvested before (−) HCMV infection or at 1, 2, 3, 5, and 7 dpi with HCMV Towne strain were subjected to RT-PCR assays. Amplified DNA fragments were separated in a 2% agarose gel and then photographed. Beta-actin gene expression was assayed for the control. **(E and F)** Towne-infected NSPC/iPSCs at 3 dpi were subjected to immunofluorescence test with anti-IE1/IE2 antibody in combination with anti-phosphorylated PERK **(E)** or anti-gB antibody in combination with anti-phosphorylated eIF2α **(F)** antibody. **(G)** Mock-infected NSPC/iPSCs were subjected to immunofluorescence test with anti-IE1/IE2 antibody in combination with anti-phosphorylated PERK (upper panel) or anti-phosphorylated eIF2α (lower panel) antibody. Nuclei were stained with DAPI. **(H)** Expression of CHOP mRNA in HCMV-infected NSPC/iPSCs. Total RNAs isolated from NSPC/iPSCs harvested before (−) HCMV infection or at 1, 2, 3, 5, and 7 dpi with HCMV Towne strain were subjected to RT-PCR assays. Representative results from three independent experiments are shown.

The unfolded protein response (UPR), induced by the accumulation of improperly folded proteins within the ER lumen (ER stress), is associated with multiple cellular responses such as neurodegeneration and apoptosis. ER stress sensor molecules, such as PKR-like eukaryotic initiation factor 2a kinase (PERK) and inositol-requiring enzyme 1 (IRE1), are activated on UPR and engage downstream signaling pathways. To examine whether the caspase-9 activation in HCMV-infected NSPC/iPSCs (Figure 3C) is associated with UPR, we analyzed phosphorylation status of IRE1α and its downstream target c-Jun NH2-terminal kinase (JNK) in immunofluorescence assays. Both IRE1α and JNK were specifically phosphorylated in HCMV-infected NSPC/iPSCs (Figure 4A and 4B), but not in mock-infected NSPC/iPSCs (Figure 4C). In concordance with the previous reports that activated IRE1α catalyzes the non-conventional splicing of the mRNA encoding X-box binding protein 1 (XBP1) [20,21], the spliced XBP1 mRNA increased gradually after HCMV infection in NSPC/iPSCs (Figure 4D). We also analyzed phosphorylation status of another sensor molecule PERK, an ER-associated serine/threonine protein kinase, and its downstream target the alpha subunit of eukaryotic initiation factor 2 (eIF2α). Phosphorylated forms of PERK and eIF2α were specifically detected in HCMV-infected NSPC/iPSCs (Figure 4E and 4F), but not in mock-infected NSPC/iPSCs (Figure 4G). The transcription factor activating transcription factor 4 (ATF4), that is preferentially translated on activation of PERK, induces the expression of C/EBP-homologous protein (CHOP/GADD153), a transcription factor with proapoptotic functions [22]. In accordance with these previous findings, the mRNA level of CHOP increased gradually after HCMV infection in NSPC/iPSCs (Figure 4H). These results suggest that UPR is involved in the activation of caspase cascade leading to apoptosis in HCMV-infected NSPC/iPSCs.

## Discussion

Important findings in this study are as follows: i) NSPC/iPSCs derived from MRC-iPS-25 cells were susceptible to HCMV infection and allow the expression of viral gene products of both early and late functions and production of infectious virions. In contrast, MRC-iPS-25 cells before induction of differentiation was either resistant to HCMV or did not support the expression of HCMV immediate-early genes; ii) the HCMV-infected NSPCs undergo apoptosis; and iii) the mechanism of the apoptosis included cytochrome c release from mitochondria to cytosol and activation of UPR-related signaling pathways.

Neuropathogenesis of HCMV infection has been studied mainly with neural cells isolated from human brain. These studies demonstrated that HCMV can infect human neural precursor cells (NPCs) isolated from fetal brains and interfere with their differentiation. Luo et al. [23] showed that HCMV infection in primary NPCs reduced the expression of Nestin, suggesting that HCMV affects the differentiation potential of NPCs. Similar results were also obtained from experiments with mouse NSCs infected with MCMV [10,13,24]. Those previous findings obtained from experiments with primary cultures of brain-derived neural cells were thus mostly reproduced in our experiments using NSPC/iPSCs. In addition, similar to the results of Odeberg et al. [8] that used NPCs derived from human brain, we also demonstrated that HCMV infection induced apoptosis in NSPC/iPSCs obtained from iPSCs. It is thus supposed that neural cells differentiated from iPSCs are a useful model to investigate neural pathogenesis of HCMV. In the human brain, NSCs are predominantly found in the subventricular region where CMV infections preferentially occur [25,26]. Analysis on the effects of HCMV infection on NSPCs can be therefore particularly relevant.

In the regulation of cellular apoptotic responses, mitochondrial dysfunction and ER stress are involved in the activation of the initiator caspase caspase-9 that functions as a trigger of cascade protease reactions leading to cell death. The finding of cytochrome c release from mitochondria to cytoplasm in HCMV-infected NSPC/iPSCs indicates that mitochondrial dysfunction is involved in the activation of caspase-9 in these cells. In addition, the demonstration of phosphorylated forms of proteins involved in UPR, including PERK, JNK, IRE1α, eIF2α, as well as that of unconventional splicing of XBP1 mRNA and up-regulation of CHOP, indicate that ER stress also plays a role in HCMV-induced apoptosis of NSPC/iPSCs. These results are in accordance with the work reported by Isler et al. [27] who demonstrated that HCMV-induced UPR in human foreskin fibroblasts. HCMV is known to encode anti-apoptotic proteins such as viral inhibitor of caspase-8-induced apoptosis (vICA) encoded by UL36 [28], and pUL38 which protects against ER stress-induced cell death by modulating the UPR pathway [29]. Our RT-PCR analysis demonstrated that such viral anti-apoptotic genes were expressed at transcription level in NSPC/iPSCs following HCMV infection (Figure 2B). Although these viral anti-apoptotic proteins did not block apoptosis of NSPC/iPSCs, they might have contributed for efficient viral replication by delaying apoptosis.

iPSCs are expected to be an innovative tool for not only regenerative medicine but also for the elucidation of pathogenesis of various diseases. Recent studies have shown that human iPSCs can be used also for modeling viral infection. Hepatocyte-like cells derived from human iPSCs were shown to be susceptible to hepatitis virus C infection and supported its replication [30,31]. Sensory neurons derived from human iPSCs were reported to be susceptible to infection with both varicella-zoster virus and herpes simplex virus [32]. While the present work was in progress, D’Aiuto and others reported on the preparation of an iPSC-derived model of HCMV infection in neural precursor cells [33]. Whereas our data described in the present study is largely consistent with their results, we further analyzed the mechanisms of apoptosis induction and elucidated the involvement of mitochondrial dysfunction and ER stress.

In conclusion, human NSPCs derived from iPSCs can be a useful model to study HCMV neuropathogenesis associated with congenital HCMV infection. They can be particularly valuable in analyzing the mechanisms of HCMV-induced apoptosis in neural cells.

## Abbreviations

HCMV: Human cytomegalovirus; iPSC: Induced pluripotent stem cell; ESC: Embryonic stem cell; NSPC: Neural stem/progenitor cell; TUNEL: Terminal deoxynucleotidyl transferase-mediated dUTP nick-end labeling; UPR: Unfolded protein response; ER: Endoplasmic reticulum; PERK: PKR-like eukaryotic initiation factor 2a kinase; JNK: c-Jun NH2-terminal kinase; IRE1: Inositol-requiring enzyme 1; eIF2α: Alpha subunit of eukaryotic initiation factor 2; CHOP: C/EBP-homologous protein; XBP1: X-box binding protein 1; IFA: Indirect immunofluorescence assay; Dpi: Days post-infection; MAP2: Microtubule-associated protein 2; GFAP: Glial fibrillary acidic protein; OSP: Oligodendrocyte-specific protein; ATF4: Activating transcription factor 4; MOI: Multiplicity of infection.

## Competing interests

The authors declare that they have no competing interests.

## Authors’ contributions

HN, HL, KM, and HA performed the experimental studies, and KI helped to analyze the data. KM, MT, HA, YM, HO, NK, and AU participated in the characterization of iPSCs and their derivatives. HN, HL, and SF wrote the manuscript. NI revised the manuscript. All authors read and approved the final manuscript.
